# Syndrome coronaire aigu du sujet jeune caucasien: à propos de 62 cas

**DOI:** 10.11604/pamj.2013.14.116.2466

**Published:** 2013-03-27

**Authors:** Bâ Hamadou, Christophe Thuaire, Grégoire Range, Thibaud Demicheli, Abdoul Kane, Franck Albert

**Affiliations:** 1 Service de Cardiologie Hôpital Laquintinie Douala, Cameroun; 2 Service de Cardiologie Hôpital Louis pasteur Chartres, Cameroun; 3 Service de Cardiologie Hôpital Général de Grand Yoff Dakar, Cameroun

**Keywords:** Sujet jeune caucasien, syndrome coronaire aigu, coronarographie, qualité de vie, young caucasian, acute coronary syndrome, coronary, quality of life

## Abstract

Chez les sujets jeunes, la survenue d'un syndrome coronaire aigu (SCA) est souvent la première manifestation de la maladie coronaire. Les objectifs de notre travail étaient de caractériser les données coronarographiques, d'étudier la survie moyenne, et la qualité de vie chez les sujets jeunes caucasiens. C'est une étude rétrospective (2000-2007) descriptive et analytique qui incluaient 62 patients d'âge inférieur ou égal à 40 ans et hospitalisés pour SCA dans le service de cardiologie de l'hôpital Louis Pasteur. Nous avons étudié les paramètres épidémiologiques, coronarographiques et le suivi évolutif moyen de 3 ans. Le sex-ratio des patients étudiés était de 7,8. La moyenne d'âge était de 34,14 ± 2,96 ans chez les femmes et de 35,02 ± 4,41ans chez les hommes. Dans 60% des cas il s'agissait d'un SCA ST-, et dans 40% des cas d'un SCA ST+. Chez 64% de nos patients, on retrouve à la coronarographie des lésions coronaires supérieures à 50% avec une prédominance des lésions coronaires monotronculaires (34%). 97% des patients ayant été traités par angioplastie ont bénéficié d'une implantation d'endoprothèses. Pendant la période de suivi 7% des patients traités médicalement, ont présenté un angor résiduel et 34% de ceux traités par angioplastie. La plupart des sujets jeunes caucasiens atteints de syndrome coronaire aigu sont de sexe masculin avec des lésions monotronculaires (type B1). Tous étaient survivants et asymptomatiques dans 2/3 des cas lors de la période de suivi.

## Introduction

Les maladies cardiovasculaires et plus particulièrement les cardiopathies ischémiques sont en constante progression dans les pays développés comme dans les pays en voie de développement [1, 2]. Chez les sujets jeunes, la survenue d'un syndrome coronarien aigu est souvent la première manifestation de la maladie coronaire. L'étude de la maladie coronaire chez les sujets jeunes présente un triple intérêt: Il y a une nécessité à préserver une population jeune, valide compte tenu du phénomène de vieillissement de la population dans les pays développés et la progression des maladies cardiovasculaires dans les pays en développement avec un âge de survenue précoce [2]. La prise en charge précoce par une stratégie de plus en plus invasive réserve un pronostic favorable à long terme. Le coût élevé de la prise en charge nécessite une accentuation de la prévention primaire des facteurs de risque cardiovasculaires dont les plus fréquents chez les sujets jeunes (tabac, obésité, dyslipidémie) sont modifiables.

Les objectifs de notre étude étaient: de déterminer les facteurs de risque cardiovasculaire d'une population jeune atteinte de maladie coronaire; de caractériser les lésions anatomiques à la coronarographie et de déterminer les modalités et les résultats de l'angioplastie coronaire chez les sujets jeunes; d'étudier la survie moyenne, la qualité de vie et le taux de sevrage tabagique post-syndrome coronarien aigu.

## Méthodes

Pour atteindre ces objectifs; nous avons réalisé une étude rétrospective, monocentrique descriptive et analytique sur une période de sept ans (2000-2007) qui incluaient les patients d'âge inférieur ou égal à 40 ans à la date de la coronarographie et hospitalisés pour syndrome coronarien aigu dans le service de cardiologie de l'hôpital Louis Pasteur de Chartres.

Les données suivantes ont été recueillies sur une fiche de renseignement: les facteurs de risque cardiovasculaires (le tabagisme persistant, l'hypertension artérielle, le diabète, la dyslipidémie, le surpoids); les données de la coronarographie (la voie d'abord, les lésions coronaires, les orientations thérapeutiques après la coronarographie); les modalités de l'angioplastie (le flux TIMI avant et après, la mise en place d'endoprothèses ou non, endoprothèses nus ou actifs, le succès ou l'échec); le suivi évolutif (le délai de suivi, la présence ou l'absence d'un angor résiduel, le sevrage ou non du tabac).

L'analyse des données s'est faite de la manière suivante: les résultats de la coronarographie sont ceux figurant sur le compte rendu définitif, établi après analyse visuelle de l'angioplasticien sur le logiciel CardioReport. Les lésions coronaires sont jugées significatives si la sténose est supérieure ou égale à 50% en cas d'atteinte du tronc commun, et supérieur ou égal à 60% en cas d'atteinte des autres artères; le suivi évolutif a été réalisé après un contact téléphonique avec le patient, un membre de sa famille ou avec son médecin traitant.

L'analyse des résultats de l'étude s'est faite à partir des statistiques de base, descriptives (moyenne, écart type, pourcentage) étant donné que l'étude concerne la description d'une seule cohorte, non randomisée.

## Résultats

Parmi les 62 patients inclus, il y a 55 hommes et 7 femmes soit un sex-ratio de 7,8. L'âge varie entre 29 et 37 ans soit un âge moyen de 34,14 ± 2,96 ans chez la femme. Chez l'homme l'âge varie de 19 ans à 40 ans avec un âge moyen de 35,02 ± 4,41ans. Le tabagisme actif est le facteur de risque cardiovasculaire le plus important de notre population (81% des patients inclus sont des fumeurs). On note chez 22,5% de patients un cumul de 3 facteurs de risques cardiovasculaires essentiellement constitués du tabagisme actif, du surpoids et de la dyslipidémie.Chez 64% de nos patients, on retrouve à la coronarographie des lésions coronaires supérieures à 50%. Il y'a dans cette population une prédominance des lésions coronaires monotronculaires (34%) siégeant surtout sur la coronaire droite et l'interventriculaire antérieure avec une répartition équilibrée.

Après la coronarographie les patients ont été répartis en deux groupes en fonction de la présence et de la sévérité des lésions coronaires: traitement médical: 71% des femmes et 45% des hommes; traitement par angioplastie coronaire: 29% des femmes et 55% des hommes.

Les patients traités médicalement se répartissent comme suit: 16% de lésions thrombotiques initiales sans sténose résiduelle significative; 11% de coronarographies strictement normales; 8% de lésions spastiques prouvées; 6% de faux positif (Myocardites). 51,61% (32 patients: 2 femmes, 30 hommes) de notre échantillon ont bénéficié d'une angioplastie coronaire transluminale percutanée. 97% des patients ayant été traités par angioplastie ont bénéficié d'une implantation d'endoprothèses dont 13% sont actifs et 84% sont nus.Un seul patient a été dilaté au ballon et avec succès. Le taux de succès primaire après angioplastie dans notre population d'étude est de 100%. On dénombre 13% des patients qui ont présenté des complications au cours des procédures de coronarographie /angioplastie représentés essentiellement par des complications mineures.

Pour le suivi les patients ont été contactés avec un recul par rapport à la date de coronarographie allant de 3 à 76 mois chez les femmes, soit un suivi moyen de 37,14 ± 21,43 mois. 4 à 84 mois chez les hommes, soit un suivi moyen de 37,51 ± 14,26 mois.

Parmi les patients traités médicalement, 93% sont restés asymptomatiques pendant la période de suivi et 7% ont présenté un angor résiduel ayant nécessité le renforcement du traitement médical. 41,5% de patients sevrés du tabac avaient été traités médicalement. Tous ces patients sont restés asymptomatiques pendant la période de suivi.

Parmi les patients ayant bénéficié d'une angioplastie 66% sont restés asymptomatiques pendant la période de suivi et 34% ont présenté un angor résiduel. Un patient parmi ceux qui avaient un angor résiduel a bénéficié d'un double pontage aorto-coronaire (IVA I-mammaire interne gauche; Marginale-mammaire interne droite), ceci après échec d'une double dilatation. 58,5% des patients sevrés du tabac avait été traité par angioplastie. Dans cette population des patients traités par angioplastie et sevrés du tabac on retrouve 25% d'angor résiduel et 75% d'asymptomatiques.

## Discussion

### Les facteurs de risques cardiovasculaires

**Le tabagisme:** Le tabagisme est le facteur de risque cardiovasculaire prépondérant chez les sujets jeunes atteints de maladie coronaire. La proportion des fumeurs varie de 76% à 91% [3–5].

*Le surpoids et l'obésité:* 50% de nos patients présentent un surpoids. Le taux est comparable à celui des nombreuses études qui retrouvent des proportions allant de 30% à 58% de la prévalence de surpoids [3, 5] chez les sujets jeunes atteints de coronaropathie.

**Les dyslipidémies:** 43,5% de nos patients présentent une dyslipidémie. Ce taux est compatible avec les proportions retrouvées par Choudhury et al [3], 12 à 89% mais est inférieur à celui de Kanitz et al [5] qui retrouvent un taux de 29,6%.

Parmi les autres facteurs de risque cardiovasculaire, on retrouve: l'hérédité (27%); le diabète (6%); l'hypertension artérielle (11%). On constate que l'hérédité, qui est un facteur de risque majeur, se retrouve dans la proportion décrite par Choudhury et al [3] (14 à 69%).

Malgré leur jeune âge, 72,5% des patients ont un cumul d'au moins de deux facteurs de risque cardiovasculaires majeurs avec le tabagisme actif qui se retrouve dans les principales associations.

### Les aspects cooronarographiques

Nous retrouvons dans notre étude 34% des patients des lésions monotronculaires (plus de 50% de la population dilatée) avec une atteinte équilibrée de la coronaire droite et de l'interventriculaire antérieure. Dans plusieurs séries, on retrouve cette prédominance des lésions monotronculaires [5–7]. Cole et al [6] retrouve des lésions monotronculaires allant de 55% à 60% dans les deux sexes avec une distribution similaire sur les deux vaisseaux principaux. Gurevitz et al [7] dans sa série de 135 femmes coronariennes âgées de moins de 50 ans retrouve 43% des patientes avec des lésions monotronculaires. Au terme de la coronarographie, 51,61%, des patients ont été jugés revascularisables par angioplastie dans notre série, compte-tenu de la prédominance des lésions de type B1 donc accessibles.

### L'angioplastie coronaire du sujet jeune

Parmi les patients dilatés, 56,25% (18/32) l'ont été sur des lésions monotronculaires et 43,75% (14/32) sur des lésions pluritronculaires. 93% des lésions dilatées sont de type B1. Le taux de succès primaires est de 100%. Kanitz et al [5] dans sa série retrouve dans la population des patients dilatés, une prédominance des atteintes monotronculaires (coronaire droite surtout puis interventriculaire antérieure) avec deux facteurs de risque cardiovasculaires majeurs. Chez les patients pluritronculaires, il retrouve 3 à 4 facteurs de risque cardiovasculaire en moyenne. Selon lui, il y aurait une apparente corrélation entre l'extension des lésions coronaires et le nombre de facteur de risque cardiovasculaire majeur par patient. Selon Morett et al [8], les patients avec plus de trois facteurs de risque cardiovasculaire ont un mauvais pronostic à long terme. 97% de nos patients ont bénéficié de la mise en place d'une endoprothèse coronaire dont 84% sont nus. Le faible taux d'utilisation des stents actifs s'explique par le fait que sur une longue période d'étude (2000 à 2002), ces stents n'étaient pas accessibles; il n'y avait pas de recommandations pour leur utilisation dans le contexte d'un syndrome coronaire aigu. Dans notre série, on retrouve 13% de complications essentiellement mineures liées à la procédure de coronarographie/angioplastie. Le taux de mortalité pendant la procédure puis à 30 jours est de 0%. Dans l'étude GUSTO IIb [9] sous branche d'angioplastie, la mortalité à 30 jours des patients de moins de 50 ans traités par angioplastie directe est de 1,1%.

### Coronarographie normale

Dans la littérature, plusieurs études sur le SCA du sujet jeune montrent [5, 10] montrent qu'il y a une population non négligeable (8 à 18% des patients) avec des coronaires angiographiquement saines. Dans notre étude, on retrouve 19% des patients avec des coronaires angiographiquement saines. Ce sont des patients SCA ST- de sexes masculins, obèses et tabagiques avec une exploration non invasive positive (épreuve d'effort ou scintigraphie myocardique). On recense actuellement plusieurs hypothèses étiologiques dans la survenue d'un SCA à coronaires saines [5, 10]:embolie coronaire, aggrégation plaquettaire, lyse spontanée du thrombus coronaire,état d'hypercoagulabilité (syndrome néphrotique, syndrome des anticorps antiphospholipides, déficit en protéine S et facteurs XII), spasme coronaire (consommation d'alcool, amphétamine, cocaïne). Dans 6% des cas, dans notre population, on retrouve des faux positifs qui sont des myopéricardites avec une présentation de SCA à ST+ à coronaires angiographiquement saines. Le registre GRACE [11], retrouve 7% de diagnostic de SCA non confirmé. Dans une série de 11 patients âgés de 17 à 39 ans masculins hospitalisés pour SCA malgré des arguments cliniques, électriques et biologiques évidents de SCA à ST+, Constantini et al [12] retiennent le diagnostic de myocardite. Ce diagnostic a été posé sur la base des arguments indirects (absence ou présence d'un seul facteur de risque cardiovasculaire, récente histoire de fièvre, patients jeunes, recherche positive d'entérovirus chez 4 patients). Huit patients qui ont bénéficié d'une coronarographie ont des coronaires saines. Constantini et al [12] concluent que le diagnostic différentiel entre SCA à ST+ et une myocardite peut être difficile voire impossible.

### Qualité de vie et sevrage tabagique

**Qualité de vie:** dans notre étude, le taux de survie est de 100% pour une période de suivi allant de 3 à 76 mois chez les femmes et de 4 à 84 mois chez les hommes. La qualité de vie est bonne dans l'ensemble de notre cohorte. En effet, 93% des patients traités médicalement et 66% par angioplastie décrivent une amélioration de leur qualité de vie et se disent asymptomatiques. Cependant après deux ans de suivi, on note 7% d'angor résiduel sans réintervention coronaire chez les patients traités médicalement et 34% (11/32) d'angor résiduel chez les patients traités par angioplastie. La revascularisation chirurgicale a été nécessaire chez un seul patient sur les 11 après échec d'une double dilatation sur les lésions de resténose coronaire. Selon Gurevitz et al [7], un angor résiduel chez un patient SCA jeune avec des lésions angiographiques significatives est un risque de survenue de décès, d'insuffisance cardiaque et de revascularisation à moyen terme.

**Le sevrage tabagique:** Selon l'étude FRAMINGHAM HEART STUDY [13], le risque relatif de survenue de maladie coronaire était trois fois plus élevé chez le jeune fumeur comparé au non-fumeur. Dans notre cohorte, on retrouve au moment du contact des patients 82% (41/50) du taux de sevrage tabagique dont 17 traités médicalement et 24 traités par angioplastie. De tous nos patients tabagiques sevrés, on retrouve seulement 14% d'angor (6/41) résiduel alors qu'il est de 77% (7/9) chez les tabagiques non sevrés. Dans l'étude EUROASPIRE [14] à 26 mois de survenue d'un SCA, on note chez ces patients un taux de sevrage tabagique de 48%, après une éducation sur les méfaits du tabac par le personnel médical. Les résultats élevés de sevrage tabagique dans notre étude peuvent s'expliquer par le fait de l'action conjuguée et permanente contre le tabac menée par le personnel médical du service de cardiologie (à travers l'unité de prévention des maladies cardiovasculaires) et l'unité d'addictologie de l'Hôpital de Chartres. En effet, tout patient fumeur porteur de cardiopathie reçoit un counselling continu sur la nécessité du sevrage tabagique avec des brochures explicatives. Après son accord, le patient est alors pris en charge dans un programme global de sevrage tabagique par l'unité d'addictologie. Pour Cole et al [6], de nombreuses études épidémiologiques prouvent une déclinaison de la mortalité chez les coronariens avec l'arrêt de la consommation du tabac.

## Conclusion

Il apparaît ainsi au terme de cette étude que la plupart des sujets jeunes atteints de syndrome coronaire aigu sont de sexe masculin. Le facteur de risque modifiable le plus important est le tabagisme. En l'absence de facteur de risque cardiovasculaire conventionnel, la recherche d'autres facteurs de risque cardiovasculaire doit être entreprise notamment la prise de pilule contraceptive (deux patientes dans notre cohorte), la consommation de cocaïne, un état d'hypercoagulabilité. Les lésions coronaires significatives sont en majorité monotronculaires et de type B1. L'angioplastie transluminale percutanée coronaire a été réalisée avec un taux de succès initial de 100%.Le pronostic était bon lors de notre période de suivi avec un taux de mortalité de 0%. La qualité de vie était bonne avec plus de 2/3 de patients asymptomatiques. Le taux de sevrage tabagique post-syndrome coronarien aigu est de 82% et paraît supérieur aux données de la littérature. Il est donc possible qu'une prise en charge multidisciplinaire de l'intoxication tabagique dès la phase aiguë permette d'améliorer le taux de sevrage tabagique à long terme. Néanmoins cette donnée nécessiterait une étude prospective pour être confirmée.

## References

[1] Thiam Massamba, Cloatre Georges, Fall Falickou (2000). Cardiopathies ischémiques en Afrique: expérience de l'hôpital principal de DAKAR. Médecine d'Afrique Noire..

[2] Wade Boubacar, Djimadou Néloum, Charles Damorou (1996). L'infarctus du myocarde chez le jeune sénégalais: étude étiologique et clinique de 14 cas. Cardiologie tropicale..

[3] Choudhury Lubna, Marsh James (1999). Myocardial infarction in young patients. The American Journal of Medicine..

[4] Grenier Olivier, Cambou Jean Pierre, Ferrieres Jean (2002). Caractéristiques initiales et prise en charge thérapeutique des sujets jeunes (âge inférieur à 45 ans) hospitalisés pour syndrome coronaire aigu: résultats des études françaises PREVENIR 1 et PREVENIR 2. Annales de cardiologie et d'angéiologie.

[5] Kanitz MG, Giovannucci SJ, Jones JS, Mott M (1996). Myocardial infarction in young adults: risk factors and clinical features. J Emerg Med..

[6] Cole JH, Miller JI, Sperling LS, Weintraub WS (2003). Long term follow-up of coronary artery disease presenting in young adults. J Am Coll Cardiol..

[7] Gurevitz O, Jonas M, Boyko V, Rabinowitz B, Reicher-Reiss H (2000). Clinical profile and long-term prognosis of women < or = 50 years of age referred for coronary angiography for evaluation of chest pain. Am J Cardiol..

[8] Morett Pierre, Gutzwiller Felix, Junod Bernard, ROSKAMM H (1981). Coronary artery disease in young adults under 35 years old: risk factors. Myocardial infarction at young age.

[9] (1997). A clinical trial comparing primary coronary angioplasty with tissue plasminogen activator for acute myocardial infarction. The Global Use of Strategies to Open Occluded Coronary Arteries in Acute Coronary Syndromes (GUSTO IIb) Angioplasty Substudy Investigators. N Engl J Med..

[10] Malek Slim, Smiri Zahreddine, Hajlaoui Nadhem (2001). Les facteurs prédictifs d'une coronarographie normale. La Tunisie médicale..

[11] Steg PG, Goldberg RJ, Gore JM, Fox KA, Eagle KA, Flather MD, Sadiq I (2002). Baseline characteristics, management practices, and in-hospital outcomes of patients hospitalized with acute coronary syndromes in the Global Registry of Acute Coronary Events (GRACE). Am J Cardiol..

[12] Costantini M, Tritto C, Licci E, Sticchi G, Capone S (2005). Myocarditis with ST-elevation myocardial infarction presentation in young man. A case series of 11 patients. Int J Cardiol..

[13] Kannel William, Mc Gee Daniel, Castelli William (1984). Latest perspectives on cigarette smoking and cardiovascular disease: the Framingham Study. J Card Rehabil..

[14] Scholte op Reimer W, de Swart E, De Bacquer D (2006). Smoking behaviour in European patients with established coronary heart disease. Eur Heart J..

